# Update on the fourth version of the Principles of Transparency and Best Practice in Scholarly Publishing: relevance for the *Korean Journal of Women Health Nursing*

**DOI:** 10.4069/kjwhn.2023.06.14

**Published:** 2023-06-30

**Authors:** Ju-Hee Nho

**Affiliations:** College of Nursing, Research Institute of Nursing Science, Jeonbuk National University, Jeonju, Korea

Journals are published in accordance with submission guidelines, which adhere to publication ethics and policies. These policies are crucial in the expanding online publishing market, as they protect readers and authors from predatory practices, including slander, and ensure transparent disclosure of who publishes which materials and how [1]. Four organizations—the Committee on Publication Ethics (COPE), the Directory of Open Access Journals (DOAJ), the Open Access Scholarly Publishers Association (OASPA), and the World Association of Medical Editors (WAME)—jointly announced the Principles of Transparency and Best Practice in Scholarly Publishing. The first version was released in December 2013, and the fourth version was announced in September 2022 [2]. These principles serve as the primary evaluation criteria for significant databases such as Scopus, and publishers or journals that fail to meet these criteria are not included in their listings. The *Korean Journal of Women Health Nursing* (KJWHN) is indexed in SCOPUS, PubMed, and the Emerging Sources Citation Index (ESCI) in compliance with the relevant standards. As part of our efforts to be listed in MEDLINE and the Science Citation Index (SCI), we are committed to ensuring the transparency of our journal by adhering to the fourth version of these standards.

The introduction to Principles of Transparency and Best Practice in Scholarly Publishing states that publishers and editors are responsible for promoting accessibility, diversity, equity, and inclusivity in all aspects of publishing [2]. This is encouraged by publishing associations, publishers, academic societies, and various publication-related organizations. COPE ensures that there is no discrimination based on gender, race, or region in the research process and academic publication field, and that prejudice is eliminated in the review process [3]. Wiley also advocates for diversity, equity, and inclusivity among the editorial team, reviewers, and authors in its publishing policies [4]. KJWHN is committed to reviewing and publishing without discrimination or prejudice. Furthermore, to ensure the diversity of authors, editors, and reviewers, we have authors from various countries and editors and reviewers from the United States, Japan, Singapore, Israel, Hong Kong, and Switzerland [5].

The fourth version of the Principles of Transparency and Best Practice in Scholarly Publishing outlines 16 items pertaining to journal content, journal practices, organization, and business practices (Table 1). Each item will be discussed in detail, with a description of how KJWHN adheres to the item [2].

## 1. Name of journal

The journal’s name should be original, and it should not cause authors and readers to misunderstand the journal’s publisher or its relationship to other journals.

## 2. Websites

As website use has become more frequent, the contents of the guidelines regarding websites are more detailed in the fourth version of the Principles of Transparency and Best Practice in Scholarly Publishing than in the 3rd version released in 2018. Firstly, it was emphasized that website security must be strengthened to safeguard users from computer viruses and malicious software. To achieve this, it was declared that websites should, at a minimum, utilize “https” protocol instead of “http.” Moreover, it was asserted that websites ought to adhere to web standards and uphold the best ethical practices in content, presentation, and application. Additionally, it is essential to verify whether any information could mislead readers or authors, and to refrain from copying the website, design, or logo of other academic journals/publishers. In instances of copying, the original site must be disclosed [2]. KJWHN employs https protocol (https://kjwhn.org) and is diligently working to bolster security [5].

## 3. Publishing schedule

Adherence to the journal’s publication schedule is required except under exceptional circumstances [2]. KJWHN is published quarterly on the last day of every March, June, September, and December.

## 4. Archiving

Journals and publishers should maintain electronic backups of journals and implement long-term digital preservation plans in case publication ceases [2]. KJWHN can be accessed without barriers through Synapse (https://synapse.koreamed.org/LinkX.php?code=2102KJWHN) and the Korea Citation Index (https://kci.go.kr) and is archived in the National Library of Korea (http://nl.go.kr) [5].

## 5. Copyright

Publication copyright policies must be clearly stated on the journal’s website, and it is important to differentiate between the copyright terms of the content and the copyright of the website itself [2]. KJWHN is copyrighted by the Korean Society of Women Health Nursing, as indicated on the website and in all published articles [5].

## 6. Licensing

The website must clearly explain license information, and if Creative Commons licenses are utilized, the license terms should also be linked to the appropriate site. A licensing policy for publishing manuscripts in third-party repositories should be in place. For instance, Wiley provides an article-sharing guideline, which offers recommendations for sharing articles on personal websites, blogs, and social media [2]. KJWHN uses Creative Commons Attribution License (https://creativecommons.org/licenses/by/4.0/) and displays it on the website [5].

## 7. Publication ethics and related editorial policies

Publication ethics encompasses the ethical guidelines that authors must adhere to and the policies required for publishing. These areas include authorship and contributorship policies, handling complaints and appeals, addressing allegations of research misconduct, managing conflicts of interest, ensuring data sharing and reproducibility, providing ethical oversight, respecting intellectual property, facilitating post-publication discussions, and implementing corrections and retractions [2]. Among these, the fourth version of the Principles of Transparency and Best Practice in Scholarly Publishing particularly emphasizes handling allegations of research misconduct and policies on corrections and retractions. Addressing allegations of research misconduct involves managing whistleblowers and cases where fraudulent activities are discovered before or after publication. With regard to corrections and retractions, it is essential to establish a policy for addressing errors or ethical issues identified in the paper after publication.

In instances where errors are identified in early access data prior to official publication, they are promptly corrected online and documented. Following publication, any corrections are published as a separate paper and linked to the original paper [1]. KJWHN has made its publication ethics and related editorial policies available on its website [5].

## 8. Peer review

In the fourth version of the Principles of Transparency and Best Practice in Scholarly Publishing, compared to the 3rd version, the detailed description of the peer review process has been strengthened. The information that should be provided on the website regarding peer review includes the following: whether or not the submitted manuscript undergoes peer review, who is responsible for conducting the peer review, all policies related to the review process, the final decision-making process for the manuscript and the individuals involved, and any exceptional cases of expert review. To ensure adherence to the review timeline, the submission and acceptance dates must be indicated on the paper approved for publication. If the review process is delayed, the author should be informed of the reason and given the option to withdraw the manuscript if they so choose [2]. KJWHN adheres to relevant standards, including website posting, for its peer review policy [5].

## 9. Access

If online content is not freely accessible to everyone, such as subscription-based or paid papers, the method of access must be clearly explained, and the subscription fee must be specified if a print copy is available [2]. KJWHN can be freely accessed at https://kjwhn.org [5].

## 10. Ownership and management

Ownership and management information must be disclosed on the website [2], and KJWHN adheres to this standard [5].

## 11. Advisory body; 12. Editorial team/contact information

Journals must have an editorial board or advisory board consisting of experts in the relevant subject area, and the names and affiliations of these members must be regularly reviewed and updated [2]. KJWHN features an editorial board composed of specialists in women's health and nursing, and the information regarding its members is consistently updated and made available [5].

## 13. Author fee

Publication fees should be indicated, and information regarding waivers should be provided if an author fee waiver is available [2]. KJWHN’s author processing fee is 600 US dollars (approximately 600,000 Korean won), which is requested from the corresponding author upon manuscript acceptance [5].

## 14. Other revenue

If there is a business model or source of revenue (e.g., author fees, subscriptions, advertising), it should be stated on the website [2]. KJWHN is financially supported by a grant from the Korean Federation of Science and Technology Societies, which is funded by the Korean government (Ministry of Science and ICT), the Korean Society of Women Health Nursing, and the authors' article processing charges [5].

## 15. Advertising

Journals must specify whether or not they publish advertisements [2]. Currently, KJWHN does not feature advertisements for products or services [5].

## 16. Direct marketing

All marketing activities carried out on behalf of journals, including manuscript submissions, must be accurate and appropriate [2]. Invitations to submit a manuscript for KJWHN are typically extended to presenters at conferences, seminars, or workshops if the topic aligns with the journal's aims and scope [5].

As previously noted, KJWHN is committed to adhering to the Principles of Transparency and Best Practices in Scholarly Publishing standards, and continuously strives to improve in accordance with the updated principles. In addition, KJWHN will continue to comply with relevant standards to guarantee transparency in journal publication and will diligently continue to communicate with our readers and potential authors.

## Figures and Tables

**Table 1.** Overview of the Principles of Transparency and Best Practices in Scholarly Publishing

Journal content	Journal practices	Organization	Business practices
Name of journal	Publication ethics and related editorial policies	Ownership and management	Author fees
Website	Peer review	Advisory body	Other revenue
Publishing schedule	Access	Editorial team/contact information	Advertising
Archiving			Direct marketing
Copyright			
Licensing			
